# Stereotactic Hematoma Aspiration Versus Standard Treatment for Brainstem Hemorrhage—Updated Meta‐Analysis, Meta‐Regression, and Trial Sequential Analysis

**DOI:** 10.1155/srat/3765769

**Published:** 2026-09-15

**Authors:** Paweł Łajczak, Ayesha Ayesha, Oguz K. Sahin, Anna Łajczak, Mable Pereira

**Affiliations:** ^1^ Medical University of Silesia, Katowice, Poland, sum.edu.pl; ^2^ Shifa College of Medicine, Islamabad, Pakistan; ^3^ Hacettepe University, Ankara, Turkey, hacettepe.edu.tr; ^4^ Lincoln American University School of Medicine, Georgetown, Guyana

**Keywords:** hemorrhage, neurological functionality, ROSA, survival

## Abstract

**Introduction:**

The standard treatment of brainstem hemorrhage (BH) remains a controversial subject, and the optimal treatment strategy remains unclear. Stereotactic aspiration of a hematoma is one of the most used techniques in BH management. This review aims to update existing knowledge on the comparison of aspiration and standard treatments for BH.

**Methods:**

This review updates a previous meta‐analysis on this subject. Four medical databases were searched from inception, and meta‐analysis was performed with a random‐effects model. Odds ratios (ORs) and mean differences (MDs) were applied for comparison.

**Results:**

A total of 15 studies were included in this review. There was a significant reduction in total mortality (OR 0.26, 95% CI 0.17–0.41, *p* < 0.001), mortality rate at 1‐month follow‐up (OR 0.32, 95% CI 0.22–0.46, *p* < 0.001), and an increased proportion of patients with mRS ≤ 3 (OR 2.69, 95% CI 1.57–4.59, *p* < 0.001) in the surgical group compared with standard treatment. Meta‐regression revealed a significant correlation between GCS at admission and total mortality. There was no significant difference in rebleeding, stress ulcers, central hyperthermia, length of hospital stay, or respiratory complications. Trial sequential analysis showed a true‐positive result for mortality.

**Conclusions:**

Stereotactic surgery is becoming a promising method for the treatment of BH. The results of this synthesis suggest that patients undergoing invasive treatment have higher survival rates and better functional outcomes compared with usual care. Although stereotactic aspiration yielded a higher survival in the meta‐analysis, quality concerns, false‐positive results for patients with mRS ≤ 3, and heterogeneity need to be acknowledged.

## 1. Introduction

The brainstem is a central nervous system (CNS) structure involved in vital functions, including breathing, regulation of blood pressure, and sleep [1]. However, these functions may be significantly impacted by brainstem hemorrhage (BH), which leads to severe neurological complications with poor functional prognosis for the patient [2].

Standard treatment of BH involves monitoring of blood pressure, managing intracranial pressure (ICP), and monitoring glucose and temperature levels, as well as addressing venous thrombosis or seizure events [3]. Although standard treatment offers a noninvasive approach for BH, it is generally considered ineffective, leading to a poor prognosis for the patient [4].

Patients with BH may be treated with various surgical strategies, ranging from heavily invasive craniotomy to minimally invasive stereotactic hematoma aspiration. Japanese researcher Takahama and colleagues first performed hematoma aspiration surgery in 1989 [5]. This type of hematoma evacuation has several notable advantages, including a minimally invasive surgery (MIS) approach, a lower risk of injury to CNS structures, and a relatively short procedure time compared with other, more invasive strategies [6].

The MISTIE III trial evaluated MIS aspiration with thrombolysis for patients with intracerebral hemorrhage [7]. This open‐label study, involving 78 hospital centers, aimed to compare surgical aspiration and standard medical management; however, the results of this randomized study did not show significant benefits of MIS, especially for hematomas with larger volumes.

The recent meta‐analysis conducted by Pustilnik and colleagues evaluated MIS aspiration and standard treatment for BH [4]. Their review, which included nine studies, demonstrated significant benefits in terms of mortality reduction and favorable neurological outcomes for patients undergoing surgery. However, significant heterogeneity in several outcomes was observed, which raises concerns about the validity of the results. Moreover, the number of studies in the literature is growing; therefore, an up‐to‐date analysis is needed to incorporate the results of the latest trials.

This meta‐analysis aims to compare existing knowledge on this subject, updating the synthesis with the most current studies. Secondly, robust sensitivity analyses will be performed, along with meta‐regression to explore factors influencing outcomes. Thirdly, as the previous review focused solely on mortality and neurological outcomes, this paper will also analyze rehemorrhage events, complications, and duration of hospitalization between the two groups. Finally, because of expected heterogeneity, the results will be validated using trial sequential analysis (TSA) to determine whether a true‐positive effect has been achieved.

## 2. Methods

The PRISMA statement was followed for study reporting, and the meta‐analysis was prospectively preregistered in PROSPERO (CRD420251057636) [8].

### 2.1. Inclusion of Studies From the Previous Review

Previous meta‐analyses included a total of nine studies [4]. This meta‐analysis incorporated eight out of nine articles. One paper was excluded [9], as a newer version of the study, conducted by the same affiliation, was subsequently published [10].

### 2.2. Inclusion Criteria

This study included pairwise or multiarm studies evaluating MIS hematoma aspiration and comparing the results to those of conservative treatment for BH. Conservative treatment was defined as the use of nonsurgical management as the primary intervention for BH

Papers with wrong types of intervention or control groups, without any outcome to run statistical analysis, published in languages other than English, with duplicate results, or abstract‐only studies were excluded.

### 2.3. Search Strategy and Databases

The search string involved terms related to BH and MIS evacuation. A detailed search strategy is available as an appendix to this study. Authors searched key medical databases, including CENTRAL, PubMed, Web of Science, and Scopus. The search was run without any search engine filters or other restrictions. Systematic search was performed from the inception to May 2025. Database files were exported into screening software.

### 2.4. Extracted Data and Outcomes of Interest

The authors extracted information regarding the number of patients in each group, the proportion of male patients, hematoma volume, the mean age of the patients, Glasgow Coma Scale (GCS) at admission, and the type of conservative management (if available). Analyzed outcomes included mortality at last possible follow‐up, mortality rate at 1‐month follow‐up, mortality at 6‐month follow‐up, rehemorrhage, the proportion of patients with modified Rankin Scale (mRS) ≤ 3 at last possible follow‐up, the proportion of patients with mRS ≤ 3 at 90‐day follow‐up, the proportion of patients with mRS ≤ 3 or Glasgow Outcome Scale (GOS) 4–5 at last possible follow‐up, length of hospital stay (days), events of central hyperthermia, stress ulcers, and respiratory tract complications.

### 2.5. Quality Assessment

Authors employed the ROBINS‐I tool and assessed seven domains, rating each from low to critical risk of bias [10].

Publication bias assessment was performed only for outcomes with at least 10 studies (in this study, only mortality), as recommended by the Cochrane Handbook for Systematic Reviews of Interventions [11]. This process involved both visual inspection of contour‐enhanced funnel plots for asymmetry and the use of Egger′s test, where *p* < 0.05 was considered statistically significant [12].

### 2.6. Pairwise Meta‐Analysis

A meta‐analysis was conducted using the “meta” package in R [13]. The Mantel–Haenszel (MH) random‐effects model with DerSimonian–Laird (DL) tau estimator was employed for binary outcomes. In contrast, the inverse variance (IV) random‐effects model with DL tau estimator was employed for continuous outcomes. Binary outcomes were analyzed using odds ratios (ORs), whereas continuous outcomes were analyzed using mean differences (MDs), both reported with 95% confidence intervals (CIs). Effect sizes were considered significant for *p* < 0.05. The *I* ^2^ index was used to assess the proportion of variance attributed to between‐study heterogeneity. Results were visualized with forest plots. Medians were converted to means when needed [14].

### 2.7. Meta‐Regression and Sensitivity Analysis

The authors have employed a leave‐one‐out sensitivity analysis to explore heterogeneity. This process involved excluding each study and exploring the reduction of *I* ^2^ or the significance of effect size. Subgroup analyses for primary outcomes (mortality and functional status) were performed based on follow‐up date. In cases of unclear or nonstandardized follow‐up, studies were classified as “other.”

Univariate meta‐regression was performed only for outcomes with at least 10 studies, as recommended by the Cochrane Handbook [11]. Covariates for the regression analysis included age, percentage of male patients, hematoma volume, GCS at admission, and proportion of patients with intraventricular hemorrhage (IVH).

### 2.8. TSA

Finally, to validate the significant outcomes in terms of true/false positive/negative effects, TSA was employed using TSA software (Copenhagen Trial Unit, Denmark) [15]. A random‐effects DL TSA was performed, with empirical settings and 80% power. An inner wedge design, O′Brien–Fleming boundaries, and a 5% Type 1 error rate were chosen.

### 2.9. Declaration of Generative AI and AI‐Assisted Technologies in the Writing Process

During the preparation of this work, the authors used Gemini Pro during writing and grammar checking process. After using this tool, the authors reviewed and edited the content as needed and take full responsibility for the content of the published article.

## 3. Results

**Figure 1:**
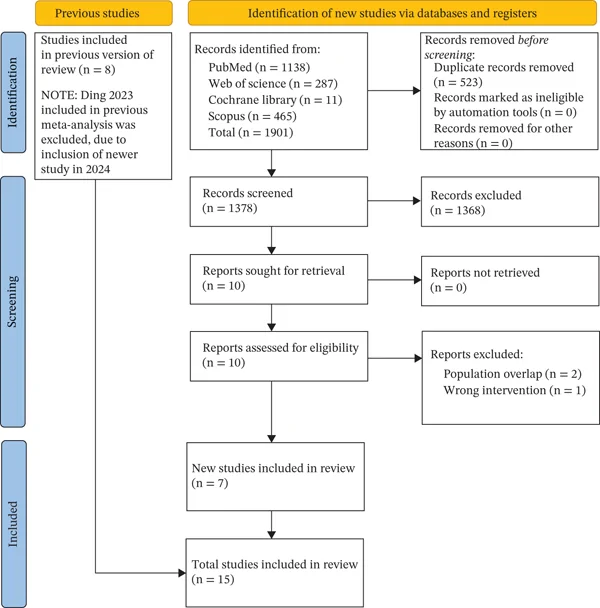
PRISMA flow diagram.

Eight studies from the previous review were initially included. Databases were systematically searched from inception. A total of 1901 articles were identified from four databases: PubMed (*n* = 1138), WoS (*n* = 287), CENTRAL (*n* = 11), and Scopus (*n* = 465). Before the screening, duplicate entries were removed (*n* = 523), and 1378 papers underwent the screening process. Ten studies were eligible for full‐text screening, and seven new studies were added. In total, this review included 15 studies [16–30]. Detailed information on the screening process is visualized in Figure 1.

This meta‐analysis included a total of 1803 patients, among whom 638 underwent surgical treatment and 1165 received standard treatment. Mean hematoma volume ranged from 6.44 to 13.7 mL. Table 1 displays the information on the included studies.

**Table 1:** Characteristics of included trials.

Study	Sample size (surgery/conservative)	Proportion of male patients (%) (surgery/conservative)	Volume of BH (mL) (surgery/conservative)	Age (years) (surgery/conservative)	IVH (%) (surgery/conservative)	GCS at admission (surgery/conservative)	Conservative treatment
Bao et al. [18]	36/46	24 (66.7)/32 (69.6)	10.79 (5.31)/7.17 (1.78)	48.98 (9.89)/50.26 (10.68)	5 (13.9)/6 (13.0)	3.39 (0.63)/3.41 (0.61)	According to AHA/ASA guidelines
Tang et al. [22] (a)	51/74	35 (68.6)/59 (79.7)	7.6 (6.5–9.7)/7.6 (5.5–9.5)	55 (49–63)/54 (44.8–61)	11 (21.6)/22 (29.7)	4 (3–5)/4 (3–5)	According to AHA/ASA guidelines: elevation of the head of the bed, lowering SBP, sedation, intubation/MV, hyperosmolar treatment, hypothermia
Xu et al. [29]	16/23	12 (75.0)/17 (73.9)	12.61 (3.64)/10.5 (3.81)	49.06 (7.46)/51.65 (8.39)	9 (56.3)/10 (43.5)	4.81 (1.42)/5.57 (1.47)	Not detailed
Zhang et al. [30]	28/25	17 (60.7)/14 (56.0)	9.0 (4.7–21.3)b/NA	53.8 (8.3)/52.6 (9.3)	6 (21.4)/4 (16.0)	6.2 (2.6)/6.0 (1.7)	Not detailed
Sun et al. [27] (a)	35/103	26 (74.3)/74 (71.8)	11.4 (9.5–13.6)/11.7 (8.5–15.9)	54.0 (46.5‐57.5)/56.0 (49.0‐64.5)	13 (37.1)/37 (35.9)	3.0 (3.0–4.0)/5.0 (3.0–6.0)	Patient confined to bed, monitoring vital signs, use of drugs to control ICP/edema, lowering BP, providing rehabilitation and moxibustion, nutritional support, prevention of complications
Ding et al. [16] (a)	96/127	63 (65.6)/78 (61.4)	10 (6.16)/11 (7.18)	56 (48.67)/57 (51.70)	30 (31.3)/22 (17.3)	6 (4.7)/6 (4.6)	Monitoring vital signs, airway protection, cardiovascular and hemostasis support, management of blood glucose and BP
Li et al. [25]	65/72	57 (87.7)/61 (84.7)	9.9 ± 5.7/12.4 ± 4.7	42.3 ± 10.5/43.7 ± 8.1	29 (21.2)	4.5 ± 2.7/4.2 ± 2.3	Airway protection, lowering BP and ICP, treatment of infections, hypothermia
Hu et al. [21]	11/243	203 (79.92)	NA	52.56 (12.43)	NA	7.99 (5.11)	Not detailed
Huang et al. [20]	11/64	59 (78.7)	6.44 ± 5.03	54.85 ± 12.69	18 (24.0)	8.73 (4.88)	Not detailed
Jang et al. [19] (b)	35/195	NA	NA	NA	NA	NA	BP and ICP control, antihypertensive therapy, hypertonic treatment
Ma et al. [28] (b)	27/30	NA	NA	NA	NA	NA	Not detailed
Wei et al. [24]	30/30	20 (66.67)/18 (60.0)	12.51 (4.40)/13.67 (5.08)	51.9 (13.4)/54.6 (11.4)	NA	NA	Bed rest, BP and ICP control, glucose control, hypothermia, nutritional support, airway protection
Yu et al. [23]	64/10	46 (62.16)	NA	54.43 (14.46)	NA	NA	Not detailed
Zhou et al. [17] (a)	113/98	175 (83)	11.3 (7.9)	49 (42–57)	125 (59)	4 (3–5)	Not detailed
Shitamichi et al. [26]	20/25	NA	6.5/10.5	57.1/59.5	NA	NA	Not detailed

*Note:* (a) indicates median with IQR; (b) indicates no exclusive data were available because of presence of other intervention/control groups, otherwise mean with standard deviation.

### 3.1. Mortality at Last Follow‐Up

**Figure 2:**
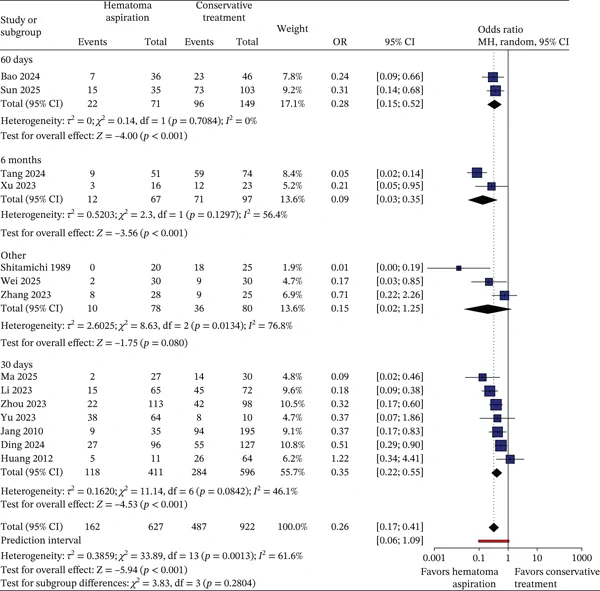
Forest plot for mortality. Subgroups for last possible follow‐ups are presented with OR and 95% CI.

There was a significant reduction of mortality in the surgical group (OR 0.26, 95% CI 0.17–0.41, *p* < 0.001, *I* ^2^ = 61.6*%*; Figure 2). Omitting Tang et al. [22] reduced *I* ^2^ to 40.4%, and effect size remained significant. There was no significant difference between mortality at different follow‐ups (*p* = 0.28; Figure 2).

**Figure 3:**
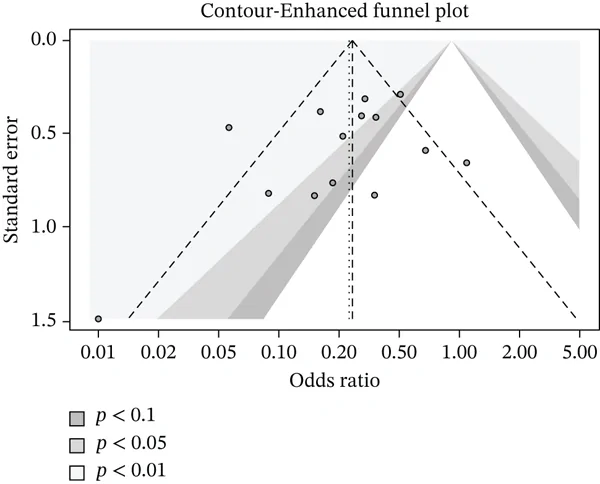
Funnel plot (contour‐enhanced) for mortality.

No visual asymmetry of the funnel plot was observed (Figure 3). Egger′s test did not indicate the presence of funnel plot asymmetry (*p* = 0.24).

### 3.2. Mortality Rate at 1‐Month Follow‐Up

**Figure 4:**
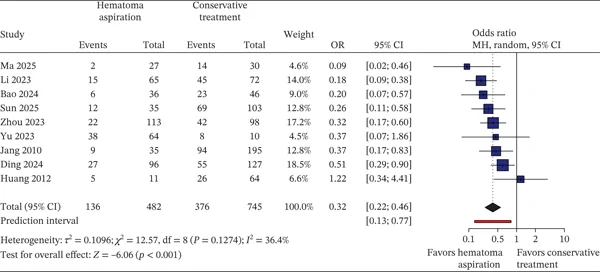
Forest plot for mortality rate at 1‐month follow‐up.

A significant reduction in the mortality rate at the 1‐month follow‐up was observed in the surgical cohort (OR 0.32, 95% CI 0.22–0.46, *p* < 0.001, *I* ^2^ = 36.4*%*; Figure 4). Omitting Huang et al. [20] reduced *I* ^2^ to 16.5%, while maintaining significance of analysis.

### 3.3. 6‐Month Mortality

For 6‐month mortality, a significantly lower mortality rate was observed in the aspiration group (OR 0.09, 95% CI 0.03–0.35, *p* < 0.001, *I* ^2^ = 56.4*%*; Figure 2).

### 3.4. Rehemorrhage

**Figure 5:**
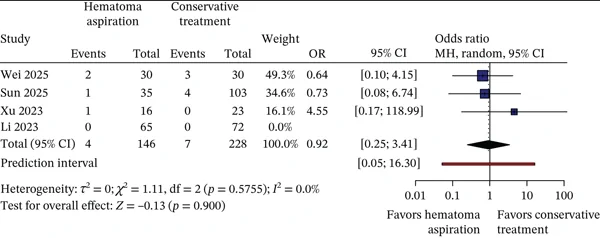
Forest plot for rehemorrhage.

There was no significant difference in terms of rehemorrhage between the two groups (OR 0.92, 95% CI 0.25–3.41, *p* = 0.90, *I* ^2^ = 0*%*; Figure 5).

### 3.5. Proportion of Patients With mRS ≤ 3

**Figure 6:**
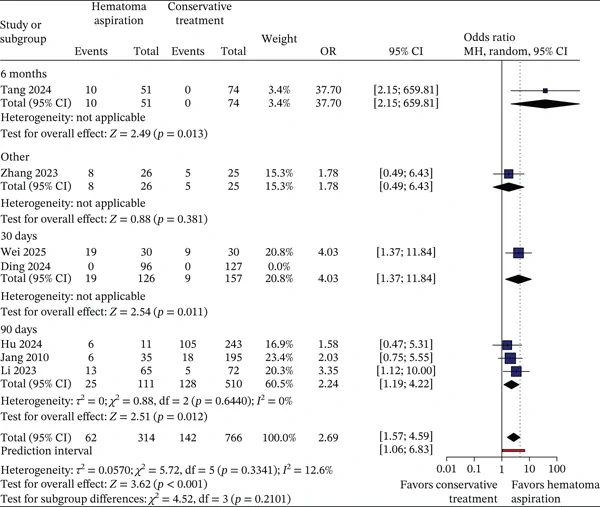
Forest plot of patients with mRS ≤ 3.

Hematoma aspiration surgery showed a significantly higher proportion of patients with mRS ≤ 3 (OR 2.69, 95% CI 1.57–4.59, *p* < 0.001, *I* ^2^ = 12.6*%*; Figure 6). The superiority of surgical group was confirmed by prediction interval (1.06–6.83). Exclusion of Tang et al. [22] reduced *I* ^2^ to 0%, while maintaining significant effect size.

### 3.6. Proportion of Patients With mRS ≤ 3 at 90 Days

At 90 days of follow‐up, the proportion of patients with an mRS ≤ 3 outcome was significantly higher in the aspiration group (OR 2.24, 95% CI 1.19–4.22, *p* = 0.012, *I* ^2^ = 0*%*; Figure 6).

### 3.7. Proportion of Patients With mRS ≤ 3 or GOS 4–5

**Figure 7:**
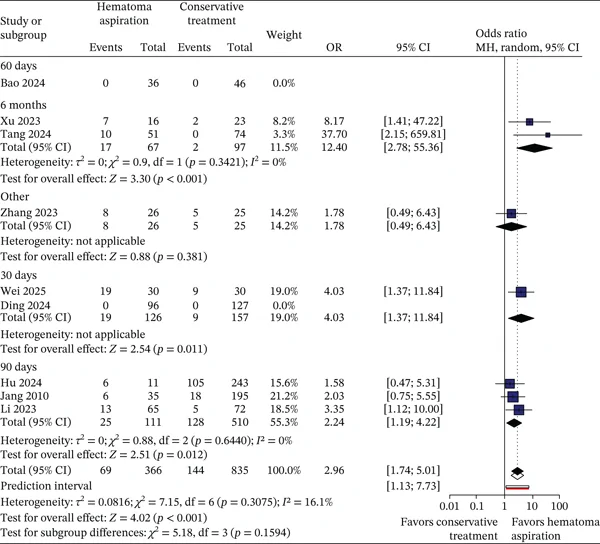
Proportion of patients with mRS ≤ 3 or GOS 4–5.

Proportion of patients with favorable outcome (mRS ≤ 3 or GOS 4–5) was significantly higher in surgery group (OR 2.96, 95% CI 1.74–5.01, *p* < 0.001, *I* ^2^ = 16.1*%*; Figure 7). Exclusion of Tang et al. [22] reduced *I* ^2^to 0%, while maintaining significant effect size.

### 3.8. Length of Hospital Stay (Days)

**Figure 8:**
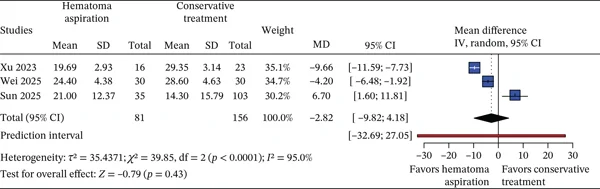
Forest plot for length of hospital stay.

The length of hospital stay was comparable between the two groups, and no significant differences were observed (MD −2.82, 95% CI −9.82 to 4.18, *p* = 0.43, *I* ^2^ = 95*%*; Figure 8).

### 3.9. Central Hyperthermia

**Figure 9:**
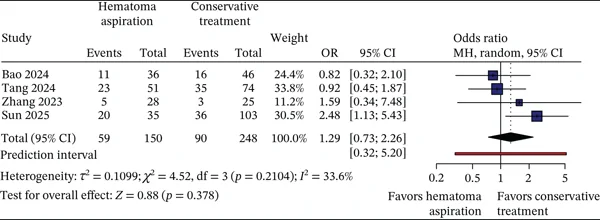
Forest plot for central hyperthermia.

The incidence of central hyperthermia was comparable between the two groups, and no significant differences were observed (OR 1.29, 95% CI 0.73–2.26, *p* = 0.378, *I* ^2^ = 33.6*%*; Figure 9).

### 3.10. Stress Ulcers

**Figure 10:**
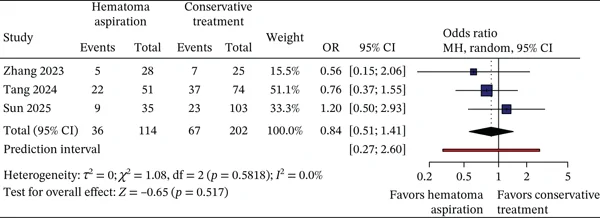
Forest plot for stress ulcers.

Similarly, no significant differences were observed in the incidence of stress ulcers (OR 0.84, 95% CI 0.51–1.41, *p* = 0.517, *I* ^2^ = 0*%*; Figure 10).

### 3.11. Respiratory Tract Complications

**Figure 11:**
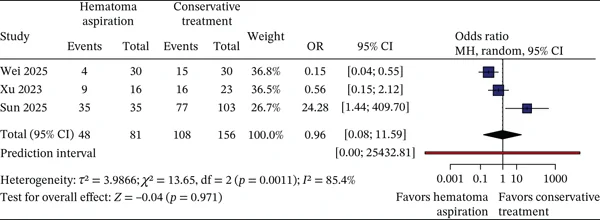
Forest plot for respiratory tract complications.

Finally, the incidence of respiratory tract complications showed no significant differences between the two groups (OR 0.96, 95% CI 0.08–11.59, *p* = 0.971, *I* ^2^ = 85.4*%*; Figure 11).

### 3.12. TSA

**Figure 12:**
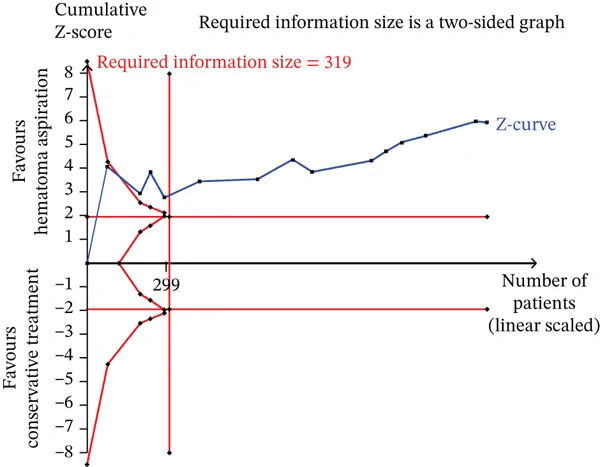
TSA plot for mortality.

The TSA performed showed true‐positive results for mortality (Figure 12), the mortality rate at 1‐month follow‐up, and 6‐month mortality. Interestingly, the number of patients with mRS ≤ 3 or mRS ≤ 3/GOS 4–5 did not reach the required sample size (resulting in false‐positive results); therefore, a definitive conclusion regarding functional outcome cannot be reached, and more patients are needed to fully understand clinical impact.

### 3.13. Risk of Bias

**Figure 13:**
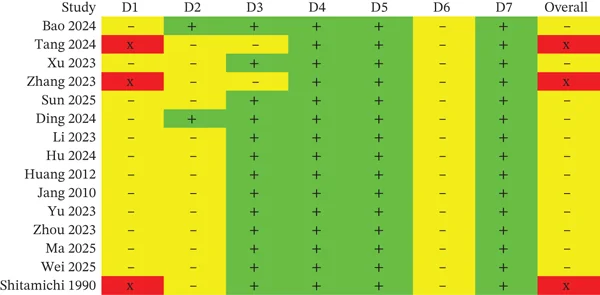
ROBINS‐I quality assessment.

The ROBINS‐I quality assessment revealed moderate concerns in 12 included studies and serious concerns in three studies (Figure 13). Most concerns were identified in bias due to confounding, measurement of outcomes, participant selection, and classification of interventions.

### 3.14. Meta‐Regression

**Figure 14:**
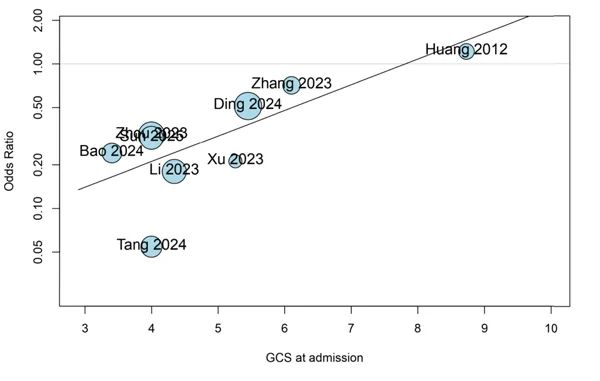
Meta‐regression bubble plot for overall mortality.

Only total mortality was eligible for meta‐regression analysis. Meta‐regression was performed to find potential correlations with mortality. Meta‐regression yielded insignificant results for age (*p* = 0.68), percentage of male patients (*p* = 0.36), hematoma volume (*p* = 0.995), or proportion of patients with IVH (*p* = 0.87). However, a significant correlation was found between the initial GCS score at admission (*p* = 0.0089, *Β* = 0.4082, standard error = 0.156, *R* ^2^ = 55.97*%*, residual *I* ^2^ = 49.97*%*; Figure 14). Clinically, this positive coefficient indicates that as the initial GCS score increases, the survival benefit of surgical aspiration relative to standard treatment diminishes.

## 4. Discussion

High blood pressure remains the most common cause of intracerebral hemorrhage, which may lead to catastrophic complications for the patients [31, 32]. The standard treatment for BH does not resolve the presence of an intracerebral hematoma, and the functional prognosis remains poor [7]. Craniotomy, although promising, did not deliver the expected improvements for patients with intracerebral hemorrhage; therefore, novel surgical strategies were needed [7].

To overcome these barriers, the number of MIS studies on hematoma has recently increased, as evidenced by the growing availability of literature. Typically, MIS aspiration involves CT‐based or computer‐assisted navigation imaging for planning the needle′s trajectory [33]. A burr hole is then drilled, a cannula is inserted, and aspiration is performed [34].

This review aimed to update existing knowledge of MIS hematoma aspiration for BH [4]. The results of this synthesis highlight several advantages of surgical treatment over conservative management.

Firstly, the mortality rate was significantly lower in the surgical aspiration cohort compared with standard treatment (*p* < 0.001). Moreover, we found a significant meta‐regression correlation between GCS at admission and mortality rate. MIS aspiration allows for hematoma evacuation; however, it is worth noting that this procedure does not guarantee complete evacuation of residual blood mass. Nevertheless, it minimizes the effect of residual hematoma and lowers the risk of potential intracranial pressure rises and pressure‐related complications. Novel technologies, including computer‐assisted surgery, may even improve clearance effectiveness; however, the number of studies remains limited [22]. These results are consistent with previous meta‐analyses on this subject, which have shown that the hematoma aspiration group exhibits a significantly higher survival rate [4]. Furthermore, the validity of these findings was confirmed through TSA, which yielded a true‐positive result for mortality outcomes, confirming that hematoma aspiration is an effective method.

Moreover, the clinical advantage of MIS aspiration was confirmed for both short‐term (1‐month follow‐up) and long‐term (6‐month follow‐up) mortality, as surgical groups showed significantly lower rates of death. These results were also validated through TSA, which confirmed true‐positive outcomes.

Notably, the impact on neurological outcomes among BH patients remains controversial. Although this meta‐analysis showed a significantly higher difference favoring the aspiration cohort in the proportion of patients with mRS ≤ 3, the TSA indicated a false‐positive result. This suggests that the required information size was not reached, and current conclusions should be treated as a trend toward better neurological outcomes rather than definitive conclusions, given the lack of randomized trials and the retrospective, heavily confounded nature of Asian‐based studies.

Although previous meta‐analyses focused solely on mortality and neurological prognosis, this review analyzed potential complications and hospitalization duration. However, this synthesis found no significant difference between these two groups in terms of complications (respiratory tract complications, rehemorrhage, stress ulcers, or central hyperthermia) or length of hospital stay. Given the retrospective nature of included studies, these outcomes need to be interpreted with caution.

Although this meta‐analysis provides an up‐to‐date synthesis, several limitations must be acknowledged. Firstly, the neurological functionality grading was inconsistent, and not all studies assessed neurological status using the mRS. This lack of consistency leads to problems in the reproducibility and transparency of results. We performed a sensitivity analysis, where mRS functional status was graded along with GOS; however, differences in grading methodology remain present. Secondly, some heterogeneity was observed in the outcomes, which could be attributed to various demographic and technical factors. Moreover, the number of outcomes available in the studies was limited, and studies focused primarily on mortality. In contrast, complications, duration of hospitalization, and short‐ and long‐term outcomes were reported inconsistently.

Additionally, a major limitation of this synthesis is that all included studies exclusively originated from Asian populations. This geographic restriction heavily limits the global generalizability of our findings. Furthermore, all included studies had a nonrandomized methodology, leading to confounding and methodological biases. ROBINS‐I assessment revealed several concerns regarding patient selection, confounding, measurement of outcomes, and more. Randomized controlled trials are needed to fully validate the outcomes of this review.

Furthermore, the concept of “standard treatment” (usual care) varied across the included studies, leading to methodological heterogeneity and differences in outcomes. AHA/ASA guidelines remain the major global treatment protocols, focusing on hyperosmolar therapy, blood pressure lowering, intubation, and more. However, given the Asian‐based nature of studies, these protocols may be different, depending on national recommendations. Therefore, the authors assume that this was a major confounding factor regarding the peristroke care, impacting survival among BH patients. A very high heterogeneity in the length of hospital stay confirms these concerns, which reflects differences in discharge protocols and care.

Finally, a false‐positive result for neurological outcome raises the questions about clinical validity, as more trials are needed to verify this outcome

## 5. Conclusions

Stereotactic surgery is a promising treatment strategy for BH patients. The results of this study suggest higher survival rates compared with usual care. However, functional outcomes after stereotactic aspiration remain unclear, as current evidence yields false‐positive results in TSA. Given the retrospective nature of the included studies, confounding biases, and the Asian‐based geography of included studies, these results need to be interpreted with caution and further validated.

## Author Contributions

All authors contributed to this manuscript. P.Ł. was a lead investigator. P.Ł. and A.Ł. performed screening. A.A., M.P., and P.Ł. performed data extraction. A.Ł. and O.K.S. performed the risk of bias assessment. All authors participated in manuscript writing.

## Funding

No funding was received for this manuscript.

## Disclosure

All authors reviewed the manuscript and approved it for final publication.

## Ethics Statement

The authors have nothing to report.

## Consent

The authors have nothing to report.

## Conflicts of Interest

The authors declare no conflicts of interest.

## Data Availability

Data sharing is not applicable to this article as no datasets were generated or analyzed during the current study.

## Appendix A Search Strategy

(((pontine OR brainstem) AND (hematoma OR hemorrhage OR haematoma)) AND (aspiration OR evacuation OR puncture OR drainage))
